# The Effect of Adding V and Nb Microalloy Elements on the Bake Hardening Properties of ULC Steel before and after Annealing

**DOI:** 10.3390/ma16041716

**Published:** 2023-02-18

**Authors:** Afshin Ghanaei, Hossein Edris, Hossein Monajati, Bejan Hamawandi

**Affiliations:** 1 Advanced Materials Research Center, Department of Materials Engineering, Najafabad Branch, Islamic Azad University, Najafabad 85141-43131, Iran; 2 Department of Materials Engineering, Isfahan University of Technology, Isfahan 84156-83111, Iran; 3 Department of Mechanical Engineering, Ecole de Technologie Supérieure, 1100 Notre-Dame Street West, Montreal, QC H3C 1K3, Canada; 4 Department of Applied Physics, KTH Royal Institute of Technology, SE-106 91 Stockholm, Sweden

**Keywords:** bake hardening, yield stress, UTS, pre-strain, annealing temperature

## Abstract

Bake hardening (BH) is a vital part of special steel production. Studies in this field have focused on steels under homogeneous yielding, but until now, none have been conducted on the phenomena that occur for steels under heterogeneous yielding. In the current study, the effect of adding Nb and V alloying elements on the strength of ultra-low carbon (ULC) steel after bake hardening was investigated. The effects of pre-strain, grain size, and recrystallization annealing temperature were analyzed, as well as the effect of Nb and V on the yield stress caused by the bake hardening process. For this purpose, five types of alloys with different V and Nb contents were melted, cast in an induction furnace, and subjected to hot hammering and hot rolling. Then, cold rolling was applied to the samples by ~80%. To eliminate the effects of cold working, tensile samples were subjected to recrystallization annealing at 750 and 800 °C for 30 min, and the samples were quickly quenched in a mixture of a NaCl solution and ice. The annealed samples were subjected to a pre-tensile strain in the range of 2–12% and then aged in a silicone oil bath at 180 °C for 30 min. Then they were subjected to a tensile test. The obtained results showed that with the increase of the pre-strain and the annealing temperature, the values of baking hardness increased. The presence of V in the composition of steel reduced the annealing temperature.

## 1. Introduction

Considering the problem of energy and fuel consumption, the automotive industry has taken steps towards the production of automobiles with low fuel consumption. The heavier the automobile, the more fuel it consumes [1,2,3]. Thus, the lightness of manufactured automobiles is an important issue in automotive factories. Therefore, automotive manufacturers in the world are thinking of producing automobiles in which the steel sheets used in the automotive bodies are optimal, both in terms of strength and lightness [4].

Due to having various features, such as a relatively low production cost, and a wide range of mechanical properties ranging from soft to very hard, steel sheets have been widely used in the manufacture of automotive components. Despite the increasing development of lighter materials, steel sheets are still considered the most important consumables in the automotive industry due to their properties such as corrosion resistance with the use of coating, high energy absorption capacity during collisions, high work hardening rate, aging ability, and excellent paintability [5,6]. Although materials such as aluminum, magnesium, and plastics have been implemented in the manufacture of car parts to reduce their weight, high-strength steels in the form of sheets are the most important option due to their wide range of strengths, chemical compositions, and surface qualities [4,7,8,9]. If high-strength steel sheets are used in the construction of an automotive body, it is possible to use sheets with a lower thickness in the construction of the automotive body without reducing the resistance to indentation, which is considered one of the determining parameters of automotive safety, and as a result, the weight of the automobile can be significantly reduced. In addition to having a high strength, the steel used in the manufacture of automotive components must also have suitable formability [10]. The formability of steels used in the automotive industry is especially important in the press workshop. Today, about 30–40% of automotive bodies are made of high-strength steel. On the other hand, with the use of this type of steel, formability, which is one of the main requirements of the automotive industry, decreases and it becomes difficult to make parts with complex shapes. Therefore, the need for high strength along with excellent formability has led to the production of new types of steel that can achieve both of these characteristics by controlling the strengthening mechanism and microstructure obtained in them. One of these types of steel is paint-hardening steel, which has been widely used in automotive manufacturing, along with steels such as dual-phase steels and transformation-induced plasticity [11,12].

Progressive steels, such as HSS and AHSS, have very good structural properties, toughness, fatigue, plasticity, and strength [13,14,15,16]. The difference between these steels and other steels is their microstructural characteristics. HSS steels are single-phase ferritic steels with a reduced pearlite content and AHSS steels are multiphase steels containing more ferrite and pearlite phases. AHSS steels contain a mutual mixture of two or more phases formed by ferrite, bainite, martensite, and residual austenite. Grain size refinement, solid solution enhancement, and precipitation enhancement serve as strengthening aids for HSS and help in phase transformation. High-strength low-alloy steels (HSLA) with a carbon content of 0.04–0.25% by weight and a manganese content of 0.7–2.0% by weight are a special group of HSS. Steels that are used for bending and welding have a carbon content of less than 0.12% by weight. The base of these steels is St52 (S355) grade, which is classified based on the chemical concept of C-Mn and a yield strength up to 355 MPa [17].

As a result of hardening, steels have a low yield strength before forming, and as a result, a high formability. After the completion of the forms, their yield strength increases, and the resistance to indentation is improved. To make and produce parts such as the bumper, hood, trunk lid, and external parts of the door, you can use sheets with less thickness and in this way reduce the weight equivalent to 7 kg in the body of each automobile. The strength of parts of the automotive body in the forming stage is due to a relatively small change in its shape, and the increase in strength due to its hard work is insignificant. In the process of curing the color, it increases significantly.

BH steels are usually designed for large press sheets of external panels, which require a high stability for deep drawing. The main advantage of BH sheets is their low yield strength and high ductility parameters before deep stretching. The most basic strengthening effect of the BH process is related to hot mechanical aging, which controls the dislocations created during deep drawing by releasing carbon and nitrogen atoms. The artificial aging process is controlled by the thermal activation of carbon atoms dissolved in the deformed ferrite and subsequently during dye curing due to the diffusion motion that occurs around the dislocations, forming Cottrell atmospheres.

The second effect is caused by the precipitation hardening of carbon atoms in ε-carbides or the formation of Fe_32_C_4_ carbides; this effect is achieved through carbon or nitrogen atoms. The diffusion of nitrogen in ferrite occurs at a high rate at room temperature, resulting in rapid material aging. For this reason, it is not possible to use a solid solution strengthened with nitrogen atoms [18].

In the current study, the effect of adding Nb and V alloying elements on the strength of low-carbon steel after bake hardening was investigated. The effects of pre-strain, grain size, and recrystallization annealing temperature were studied, as well as the effect of Nb and V on the yield stress caused by the bake hardening process.

## 2. Materials and Methods

### 2.1. Casting and Homogenization

For alloying and casting, an induction furnace with a medium frequency and a capacity of 30 Kg made by the German company INDUCED GOLLINGEN VEB was used. The ULC steel rods were melted in the furnace under argon gas conditions at 1570 °C and after deoxygenation, 0.03–0.09% ferro-vanadium and ferro-niobium were added to the melt. The recovery rate for both alloys was 70%. In each step of adding ferro-niobium to the melt, ~5 min were allotted for it to dissolve. After that, the melt was poured into the crucible and then into the ceramic molds. The dimensions of the cast blocks were 20 mm × 50 mm × 250 mm. The analysis of primary and cast steels was determined by an ARUN METAL SCAN quantometer device made in England. The analysis of the chemical composition of samples is shown in Table 1. After casting, the samples were homogenized at 1200 °C for 1 h.

### 2.2. Hot/Cold Rolling

The samples were subjected to hot rolling using a two-high rolling machine (model 2/4 RM-125D, England; diameter of working rolls: 125 mm). The rotation speed of the rollers was chosen as 5 m/min and strain rate was 0.27 s^−1^. First, the samples were preheated at 1250 °C for 30 min and finished temperature was 970 °C. Then, during two passes, a 25% reduction in thickness was applied to the samples in each pass. Subsequently, the samples were quickly quenched in cold water. Then the samples were subjected to cold rolling until an 80% thickness reduction was achieved.

### 2.3. Annealing

Recrystallization annealing was done at 650, 750, and 800 °C under an argon gas atmosphere for 30 min and then the samples were quickly cooled in a mixture of NaCl solution and ice.

### 2.4. Hardness

The hardness of the samples was measured by an Otto Wolpert-Werk GmbH hardness tester by the Vickers method (HV30). Hardness was measured 5 times for each sample and its average was recorded.

### 2.5. Tensile

Tensile samples were prepared in a rolling direction and according to the ASTM-E8 sub-size standard. First, the samples were subjected to pre-tensile strains in the range of 2–12% and with a strain rate of 0.4 × 10^−4^ s^−1^ by the Hunsfield H50KS tensile machine made in England. After applying a certain amount of pre-strain, the force was stopped and the samples were subjected to the BH test in the next step. After the BH test, the samples were again loaded up to the fracture with the mentioned strain rate. To determine the yield stress increase during the BH process, the final yield stress of the samples as well as the flow stress after the pre-strains were determined.

### 2.6. Bake Hardening

After applying the pre-strains, to perform the BH test, the samples were aged in a silicone oil bath at 180 °C for 30 min.

### 2.7. Characterizations

The samples were ground using 60, 200, 400, 800, 1200, and 1500-grit papers silicon carbide sandpaper and polished with 3 μm alumina solution. The etching solution used in all cases was 2% Nital solution and Marshall solution. Marshall solution contains an equal proportion of pre-made solutions A (8 g of oxalic acid, 5 cc of sulfuric acid, and 100 cc of distilled water) and solution B (30% hydrogen peroxide). First, the samples were etched in Nital solution for 5 s, then immediately etched in Marshall solution for 4 s. The microstructure of the samples was examined by Nikon Epiphot 300 optical microscope and Leo 435 VP scanning electron microscope. The average size of grains was determined by ImageJ software, v1.52a.

## 3. Results and Discussion

### 3.1. Microstructure of Samples after Hot Rolling

**Figure 1:**
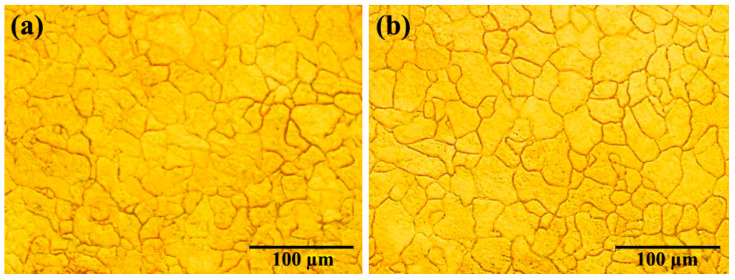
OM micrographs of hot-rolled samples: (**a**) sample B and (**b**) sample E.

The OM microstructure of samples B and E after hot rolling is shown in Figure 1. The grain size of sample E (Figure 1b) is equal to 35.3 µm and the grain size of sample B (Figure 1a) is equal to 30.6 µm. One of the reasons why sample B is finer than E is the amount of Nb present in the two samples. The amount of Nb in sample E is 0.007%, while the amount of Nb in sample B is 0.031%. Nb has played a very important role in refinement [19]. The results of the hardness and tensile tests of samples B and E can be seen in Table 2. The strength and hardness of sample B are higher than those of sample E. Since both underwent hot rolling under the same conditions, this difference is caused by the difference in the amounts of the Nb and V microalloy elements and their strengthening mechanisms.

### 3.2. Microstructure of Samples after Cold Rolling

**Figure 2:**
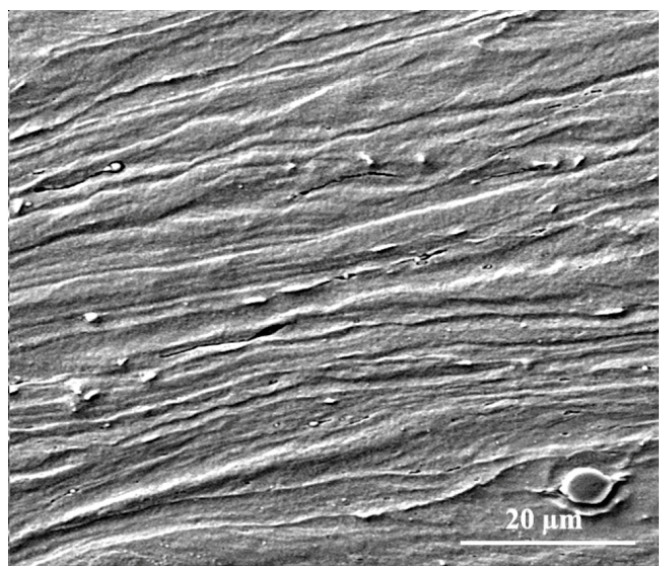
SEM micrograph of cold-rolled sample D.

The SEM micrograph of the cross-section of sample D in the rolling direction is shown in Figure 2. As can be seen, due to the cold work, the ferrite grains are elongated in the rolling direction. The results of the hardness and tensile tests on the cold-rolled samples are shown in Table 2. The comparison of the results obtained after hot rolling and cold rolling shows that applying cold work increased the hardness and strength and reduced the elongation. With the increase in crystal defects, including dislocations due to high plastic deformation (~80%), the internal energy in cold rolling increased and caused the instability of the structure in terms of thermodynamics [19]. As seen in Table 2, sample B has the maximum tensile strength and its hardness value is higher than that of the other samples. It is interesting to note that sample B has the maximum elongation.

### 3.3. Microstructure and Mechanical Properties

**Figure 3:**
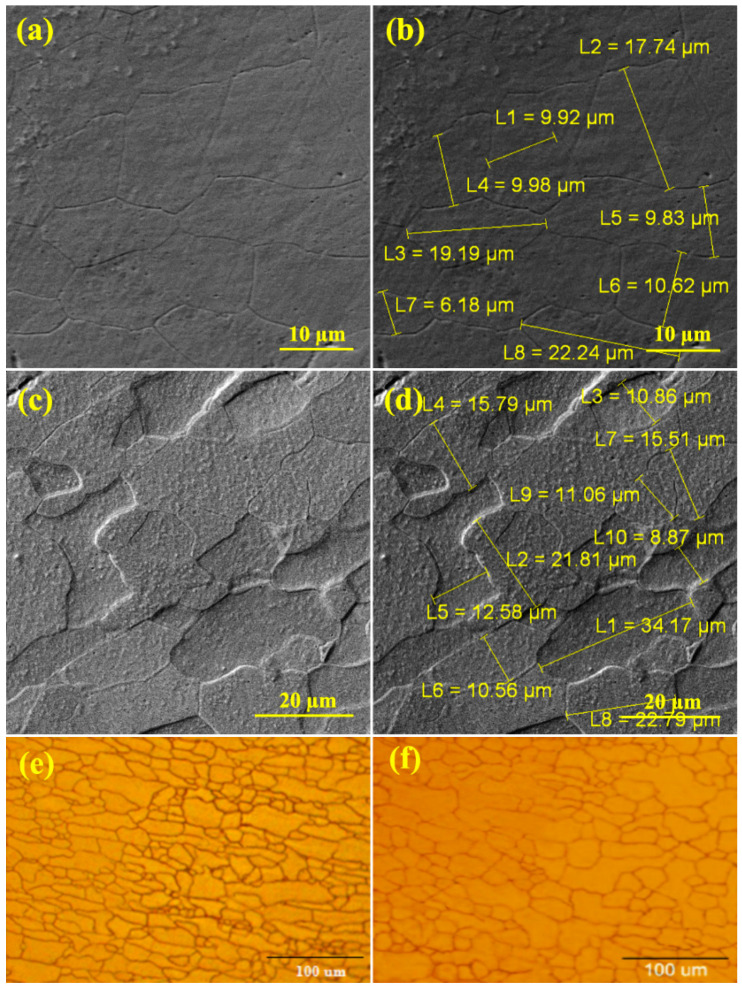
SEM and OM micrographs of sample A at different annealing temperatures: (**a,b,e**) 650 °C and (**c,d,f**) 750 °C.

The SEM and OM micrographs of sample A after different annealing temperatures are shown in Figure 3. As can be seen, the recrystallization annealing eliminated the effects of cold rolling in the microstructure. The ferrite grains, which were elongated in the rolling direction due to cold work, have turned into coaxial grains. The recrystallization temperature of ferritic-pearlite steels that are continuously annealed is lower than the eutectoid temperature and in the range of the two phases of ferrite and cementite. Applying severe cold work fractures the pearlite layers and turns them into small particles. As can be seen from the microstructure of sample A, at the annealing temperature of 650 °C (Figure 3e), the grains are elongated. Therefore, complete recrystallization is not seen at 650 °C. At 750 °C, recrystallization is complete (Figure 3f). The grains sizes at 650 °C and 750 °C were 24.9 µm and 37.28 µm, respectively. Due to the absence of the Nb microalloy element in sample A, there was an increase in grain size.

**Figure 4:**
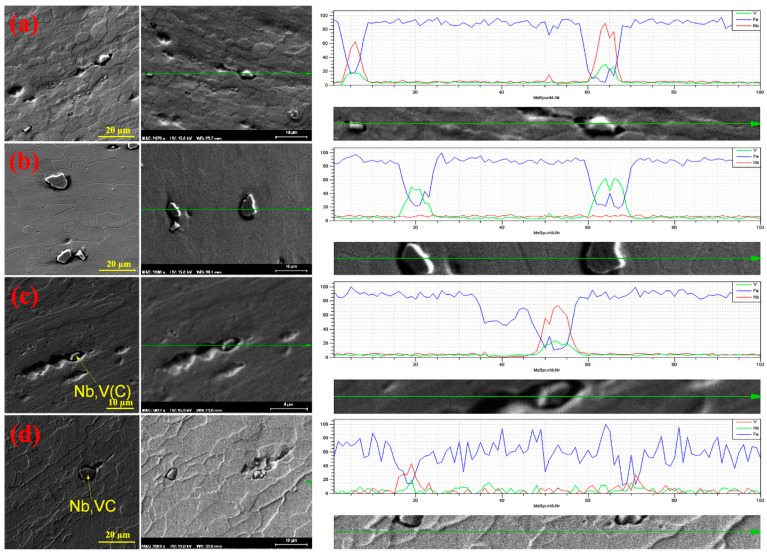
SEM micrographs and EDS analysis of the annealed samples: (**a**) sample B, (**b**) sample C, (**c**) sample D, and (**d**) sample E.

**Figure 5:**
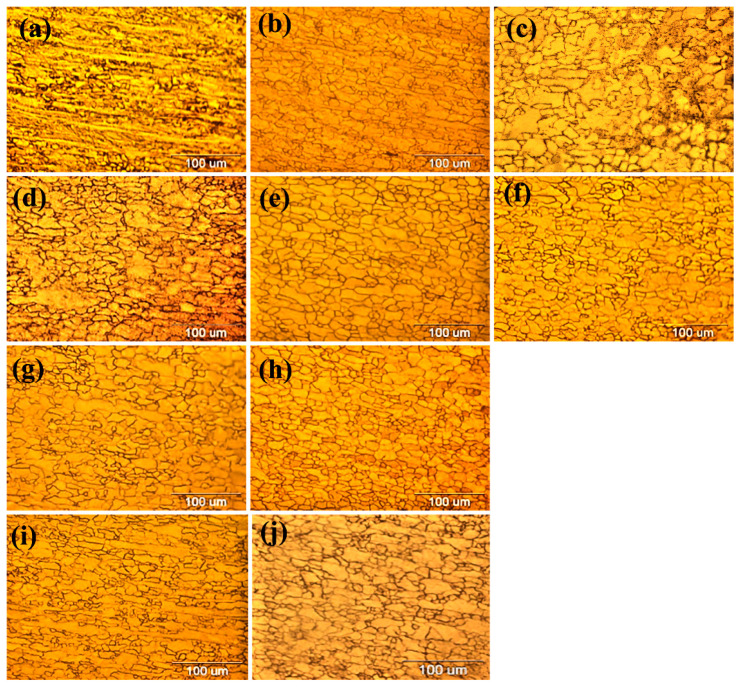
OM micrographs of the samples of the annealed samples at different temperatures: (**a**) sample B at 650 °C, (**b**) sample B at 750 °C, (**c**) sample B at 800 °C, (**d**) sample C at 650 °C, (**e**) sample C at 750 °C, (**f**) sample C at 800 °C, (**g**) sample D at 650 °C, (**h**) sample D at 750 °C, (**i**) sample E at 650 °C, and (**j**) sample E at 750 °C.

Figure 4a and Figure 5a–c show the microstructure of sample B after different annealing temperatures. It can be seen that the grain structure is elongated in the rolling direction at the annealing temperature of 650 °C and complete recrystallization is not achieved. At a temperature of 750 °C, recrystallization is achieved in a small amount, but most of the grains are stretched in the direction of rolling. Also, the grain structure is not completely aligned at the annealing temperature of 800 °C. Therefore, due to the high weight percentage of Nb and V in sample B’s steel, the temperature of 800 °C is not the complete recrystallization temperature of this steel. By performing an EDS analysis, it was found that the precipitate formed was a Nb–V carbide complex and its size was 6.77 µm.

Figure 4b and Figure 5d–e show the microstructure of sample C after different annealing temperatures. In sample C, the grain structure is often polygonal ferrite. At the temperature of 750 °C, the grain structure is more concentric, and what is clear from the image indicates that complete recrystallization is achieved at a temperature of 750 °C. Because the amount of V in this sample is more than the amount of Nb, most of the precipitates are (V,C) precipitates. After the treatment, the Nb(C,N) carbonitride was immediately quenched with water, therefore it was observed as undissolved Nb(C,N).

**Figure 6:**
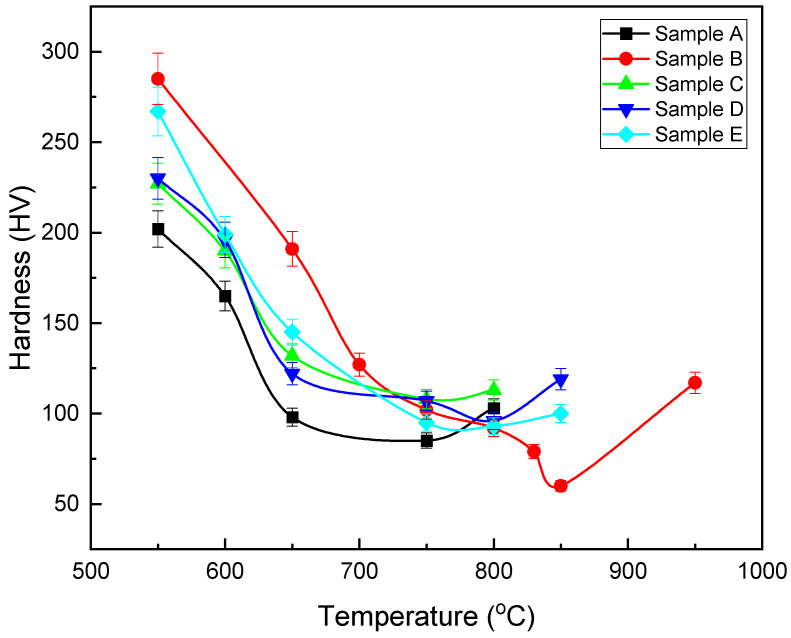
Hardness versus annealed temperatures of the different samples.

In sample D, the grains are aligned and uniform at 750 °C, which can be considered as the complete recrystallization temperature of sample D (Figure 4c). The EDS and linear analyses were used to identify the precipitate type: Nb,V(C) complex precipitates. In sample E, the structure of the grains at the annealing temperature of 750 °C, the elongation of the grains in the rolling direction decreased, but the recrystallization process was not completed. Therefore, the temperature of 750 °C is not the complete recrystallization temperature of this sample. According to Figure 4c, the precipitate size of the formed complex was equal to 1.96 µm. Figure 4d shows the distribution of the Nb and V microalloy elements in sample E. The size of the precipitate of the Nb complex V(C) measured was equal to 7.4 µm. The heterogeneous precipitation of carbide (Nb,V)C at the interfaces of undissolved large particles (Nb,V)(C,N) was dominant in the temperature range of 850–1000 °C in all the samples without strain. Previous studies have stated that insoluble Ti-rich precipitates can act as preferential nucleation sites for freshly deposited Nb-rich carbides in V-containing microalloy steels [20]. The distribution of microalloy elements has been carried out in the same way. The resulting hardness changes according to different annealing temperatures are shown in Figure 6. According to the figure, it can be seen that the minimum hardness is obtained at 750 °C and the hardness increases after a temperature of 750 °C. The reason for that is the complete recrystallization after the temperature of 750 °C.

The tensile test causes the crystal’s deformation and creates numerous defects in it, which are considered as preferential sites for heterogeneous nucleation. During rapid cooling, it is not possible to directly observe heterogeneous precipitation on the crystal’s defects. According to results of heterogeneous precipitation on the defects in crystal in austenitic steels [20,21,22], it can be found that fine (Nb,Ti,V)C carbides randomly distributed on crystal defects (subgrain boundaries and dislocations) postpone austenite recrystallization. According to previous research [23] on austenite, strain causes deposition in subgrain boundaries and dislocations, which leads to the postponement of austenite’s recrystallization. Based on the results of the embryo radius and Gibbs free energy changes calculations, Okaguchi et al. [24] reported that during nucleation in a Nb–Ti microalloyed steel, precipitation occurs mainly at subgrain boundaries and dislocations. Former austenite grain boundaries can also be considered as nucleation sites for precipitation. Nevertheless, in precipitation resulting from strain, nucleation occurs preferentially in subgrain boundaries and dislocations [25,26,27,28]. According to the reports of Hansen et al. [25], the strain causes precipitation first in the deformation bands and grain boundaries of austenite and then in the substructural features in non-crystallized austenite, which causes the postponement of austenite recrystallization. The studies of Kwon et al. [26] showed that in deformed austenite, nucleation can occur in prior austenite grain boundaries, as well as subgrain boundaries and dislocations. Nevertheless, in the current study, regardless of the deformation temperature, it was difficult to observe the grain boundary precipitates, and therefore it can be concluded that at 850 and 900 °C, grain boundary precipitation is insignificant compared to subgrain boundaries and dislocations [20,29].

The acceleration of precipitation kinetics at strains lower than 900 °C is due to the precipitation of (Nb,Ti,V)C carbides in crystal defects, such as subgrain boundaries and dislocations, as well as the precipitation of deformed (Ti,Nb)(C,N) particles at the interface [20].

In previous studies, it has been stated that by changing the element-induced formation of carbide, the parameter of the carbide network can be changed, which can decrease or increase the parameter of misfit with the matrix. Therefore, the interfacial structural energy of the matrix/carbide changes. On the other hand, the continuity of carbidizing elements reduces the contribution of chemical bonding energy to surface energy, which is a factor that reduces the surface chemical energy [30].

**Figure 7:**
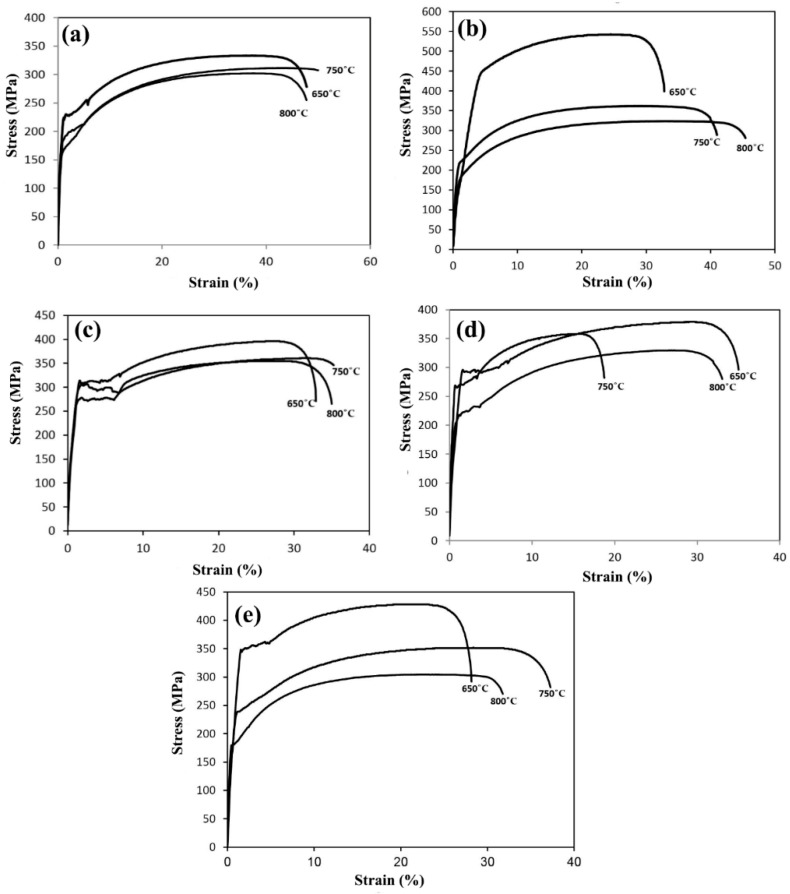
Engineering stress–strain graphs of the samples at different annealed temperatures: (**a**) sample A, (**b**) sample B, (**c**) sample C, (**d**) sample D, and (**e**) sample E.

For further investigation, engineering stress–strain diagrams for all three temperatures are shown in Figure 7. As seen in the figure, sample B has the highest tensile strength and yield stress at an annealing temperature of 650 °C. As the annealing temperature increases, the total elongation increases. Additionally, with the increasing annealing temperature, the grain size increases. The results of the stress–strain diagrams are shown in Table 3. For sample B, the tensile strength and yield stress decrease with the increasing annealing temperature, and the grain size increases with the increasing temperature. Considering the high amount of the Nb microalloy element in sample B compared to that of the other samples (0.031%), the finer grain of sample B is not far from our expections and the effect of the Nb present in the finer grain of sample B can be justified. With the increasing temperature, the tensile strength decreases and the grain size increases. But the amount of yield stress decreases with the increasing temperature up to 800 °C. This increase in yield stress is caused by the increase in dissolved carbon in the system [31]. For sample D, with the increasing annealing temperature, the tensile strength and yield stress decrease and the total elongation increases. For sample E, with the increasing recrystallization annealing temperature, the tensile strength and yield stress decrease and the total elongation increases. Additionally, with the increasing annealing temperature, the grain size increases. The lowest hardness obtained was recorded at 800 °C. According to Figure 7, it was observed that the tensile strength and yield stress at the annealing temperature of 650 °C were very high and indicated that the recrystallization of the grains did not start at this temperature. According to the graphs and information obtained, and considering that the samples of the five types of steel should be compared at the same annealing temperature under the same conditions, therefore, the temperature of 750 °C was chosen and the different mechanical properties of all five types were compared at this temperature. Moreover, for further investigation, the annealing temperature of 800 °C was chosen and a BH test was also conducted at that temperature. It is worth mentioning that the number of researched steel samples was limited due to the number of tests, so the tensile test was performed at two temperatures of 750 and 800 °C.

In annealed samples, factors such as the surrounding structure, the composition of the alloying elements, morphology, and size affect the stability of residual austenite [32]. Primary banded austenite and other austenites with low stability experience phase transformation at lower strains. Applying a pre-strain causes the initially banded austenite to be divided into small units; this reduces the material’s anisotropy. Numerous substructures such as stacking faults, dislocations, and subgrain boundaries are formed around austenite simultaneously [33]. The substructure formation and the effect of refining increase the austenite’s stability at a strain of 0.02 [34,35,36].

As the annealed samples are subjected to the thermal treatment, the mechanical properties and microstructures of the steel are improved. The reduction in supersaturation and dislocation density improves the steel’s ductility during the thermal treatment [37]. In these conditions, inducing pre-strain causes an increase in nucleation sites, resulting in the precipitation of several tiny vanadium carbides from the matrix [38].

Table 3 shows how much each strengthening mechanism contributes to the yield strength (YS) of the samples. When the pre-strain increases, the strengthening effects of dislocations and grain boundaries also increase simultaneously. In the pre-strain samples, precipitation strengthening, grain boundary strengthening, and dislocation strengthening have a significant contribution to the steel’s yield strength [36,39].

**Figure 8:**
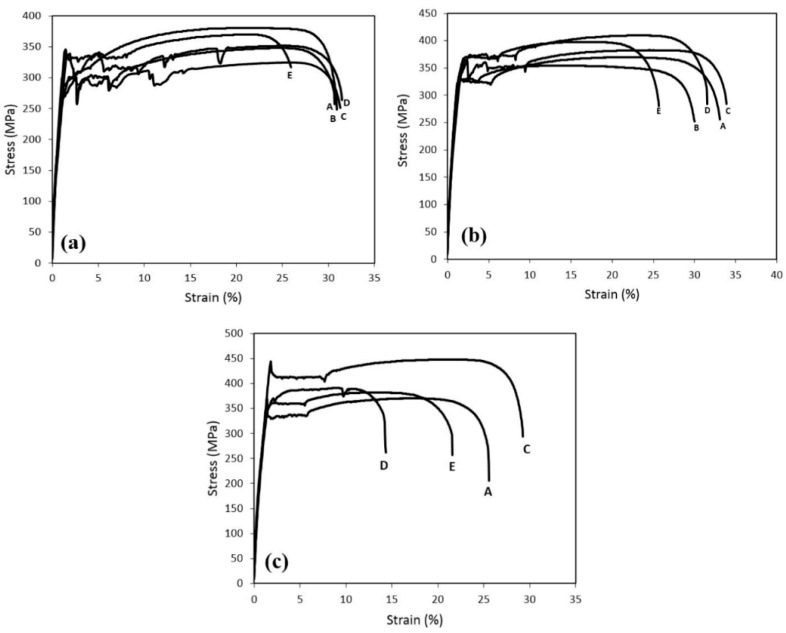
Engineering stress–strain graphs of the samples after BH treatment with different pre-strains: (**a**) 2%, (**b**) 8%, and (**c**) 10%.

The effect of the Nb and V microalloy elements on mechanical properties, as well as the stress–strain diagrams of the samples after the BH test with different pre-strains are shown in Figure 8. As shown in the figure, the highest tensile strength value was for sample B. The reason for this is the higher amount of the Nb and V microalloy elements in this steel, and due to the tendency and high potential of Nb to absorb carbon, as well as the lower dissolution temperature of V(C,N) than that of Nb, the amount of dissolved carbon after annealing is high, which causes an increase in the tensile strength. The more interstitial elements that are free in the system, the higher the tensile strength and BH. In comparing titanium-containing steels with Nb steels, it is important to note that Nb as a carbonitriding element with the formation of Nb(C,N) precipitates plays a more important role than Ti(C,N) precipitates in steel [31]. The reason is that Nb has a strong affinity to react with carbon, and the dissolution temperature of Nb precipitates is lower than the dissolution temperature of titanium precipitates, so at the applied annealing temperature, a larger volume of Nb precipitates is dissolved and causes an increase in the dissolved carbon of the system. In Figure 8a, it is observed that sample E has the highest BH value at a pre-strain of 2%. The reason is that sample E has the lowest amount of Nb (0.007%). Therefore, the most formed precipitates according to the amount of V (0.02%) are VC precipitates. Considering that the dissolution temperature of Nb precipitates is higher than the dissolution temperature of V precipitates, at the annealing temperature of 750 °C, the V precipitates, which form the largest volume of the precipitates, start to dissolve first and increase the dissolved carbon of the matrix. The type of dominant mechanism that increased the strength during the process was caused by two factors. The first factor is the increase in the density of dislocations and the second factor is the locking of dislocations by the diffusion of interstitial atoms [40]. Therefore, it seems that the difference in the type of behavior and properties of BH in the five types of samples is due to the competitive performance of the above two factors. After annealing, sample E contained the highest amount of interstitial soluble elements in the system. Therefore, a lower percentage of elements were precipitated in this steel sample compared to that of the other four samples. The low percentage of precipitates in sample E means that the dislocation agents create dislocations with a greater speed and power [41]. Because of the amount of precipitates, which is the limiting factor for dislocation, in this sample, its amount is very low; therefore, after pre-strain, the dislocation density increased. On the other hand, due to the significant presence of interstitial elements in this sample compared to that of the other four samples, a higher percentage of dislocations in this sample are locked by interstitial elements. Therefore, despite the increase in the dislocation density, the locking of these dislocations was carried out with great intensity. This caused a significant increase in the properties of BH with a pre-strain of 2% [40]. As can be seen in sample B, with the increase in the pre-strain, the value of the final tensile strength and the value of BH increased. Additionally, the value of the yield stress increased with the increase in the pre-strain. The value of the yield stress in sample B is higher than that of sample A, and this increase is due to the presence of microalloy elements. Moreover, the length of the Lüders bands is less in this sample. In Figure 8c for sample C, it is worth considering that increasing the pre-strain as long as there is sufficient dissolved carbon in the structure will increase the BH; otherwise, the BH value will decrease with the increase in the pre-strain. As can be seen for the pre-strain of 10%, the absence of sufficient carbon to lock the produced dislocations caused a slight increase in the yield stress after the completion of the BH process. Moreover, the BH in the 10% pre-strain is the maximum. As shown in Figure 8a, the amount of Lüders strain in the 2% pre-strain was 14.5%; the reason for the high value of this band is due to the wide yield loss, which increased the Lüders strain. With the increase in the pre-strain, the Lüders strain decreased. In Figure 8a for sample D, the amount of Lüders strain in the 2% pre-strain was equal to 15.4%. The reason this band is high is due to the drop in width yielding, which increased the Lüders strain. Additionally, the highest tensile strength and the lowest yield stress are related to the pre-strain of 10%. As the pre-strain value increased from 2 to 10%, the value of the BH increased. Moreover, the maximum value of the BH in the pre-strain was 10%. In Figure 8b for sample E, in the 4% pre-strain the highest Lüders strain was achieved and this is due to the drop in the width yielding, which increased the Lüders strain. Furthermore, the 10% pre-strain had the highest tensile strength and a low yield stress. By increasing the amount of pre-strain, the density of dislocations increased and due to the low amount of carbon content, the strengthening mechanism is the formation of the Cottrell atmosphere [5]. Therefore, with the increase in the pre-strain, the value of BH the increases. The critical condition for increasing the yield stress due to the mechanism of nucleation and growth of carbide precipitates is the presence of sufficient carbon in the structure. At the pre-strain of 2%, the low density of dislocations (compared to the higher pre-strain) on the one hand and the high amount of dissolved carbon on the other hand cause the Cottrell atmosphere to be saturated in the pre stages of agin; the second stage of aging is the precipitation of carbide particles [5]. At the 2% pre-strain, the increase in yield stress due to the formation of the Cottrell atmosphere was low, due to the low density of dislocations and the low suitable sites for the nucleation of carbide precipitates. As a result, coarse precipitates were formed. The formation of fine carbide particles near the dislocations in the early stages of aging during the BH process and the growth of these particles over time has been observed [41]. Coarse precipitates will have less ability to prevent the movement of dislocations than fine and dispersed particles while applying deformations after the BH process. In addition, with the formation of precipitates in the pre-strain aging, sufficient opportunities are provided for the loss in the interaction between the precipitates’ particles and the matrix, and this causes the particles to be easily cut by dislocations during the application of stress after the BH process. For this reason, the overall yield stress increases at the 2% strain and is less than that of the subsequent pre-strains.

**Figure 9:**
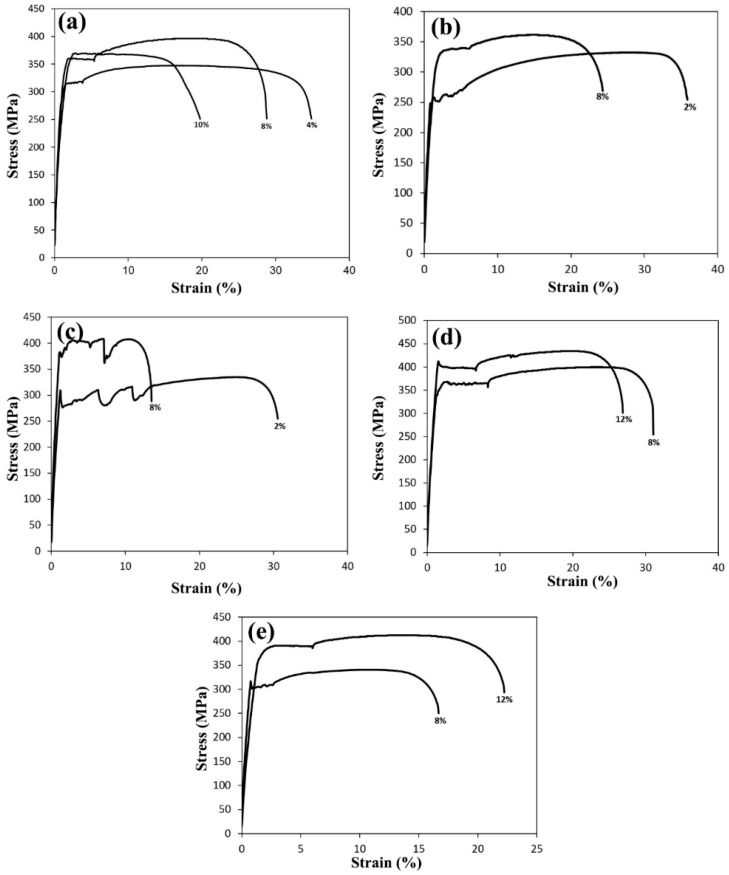
Engineering stress–strain graphs of the samples after BH treatment and annealed at 800 °C with different pre-strain: (**a**) sample A, (**b**) sample B, (**c**) sample C, (**d**) sample D, and (**e**) sample E.

To investigate the annealing temperature on the mechanical properties of the samples and the amount of BH, the samples were subjected to recrystallization annealing at 800 °C. According to Figure 9 and Table 4, it is observed that the value of the BH increased with the increase in the pre-strain and the Lüders strain value decreased. The engineering stress–strain diagrams of sample B annealed at 800 °C are shown in Figure 9b. According to the figure, the amount of yield stress in the samples with 2% and 8% pre-strain had a high difference. This difference can be attributed to the amount of dissolved carbon being low compared to the density of dislocations, and the BH value is not significant compared to that of the 2% sample. In Figure 9c for sample C, a large amount of Lüders strain is observed in the graph of the 2% sample. The reason for this is the drop in width yielding, which increased the Lüders strain by 12%. As seen in Figure 9d for sample D, the 12% sample had a distinct upper and lower yield point compared to those of the 8% sample. Additionally, the value of the Lüders strain in the diagram is 12% less than that of the 8% strain. In Figure 9e for sample E, the tensile strength and BH value increased with the increase in the pre-strain.

**Figure 10:**
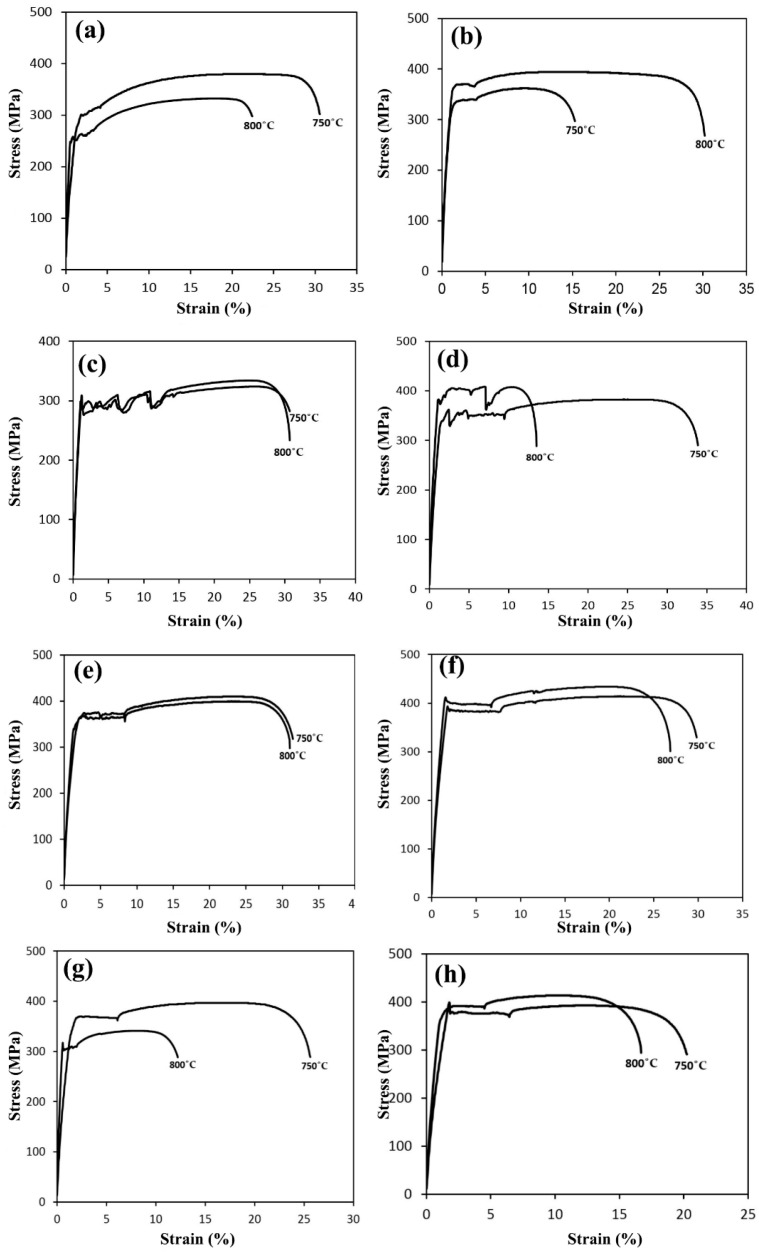
Engineering stress–strain graphs of the samples after BH treatment and annealed at 750 and 800 °C with different pre-strains: (**a**) 2% sample B, (**b**) 8% sample B, (**c**) 2% sample C, (**d**) 8% sample C, (**e**) 2% sample D, (**f**) 8% sample D, (**g**) 2% sample E, (**h**) 8% sample E.

The engineering stress–strain diagrams of the samples annealed at 750 and 800 °C and subjected to pre-strains of 2% and 8% were examined and compared. According to Figure 10, it is observed that in sample B, with the increase in the annealing temperature, the value of the BH increased from 25 to 51 Mpa. Considering the amount of the Nb and V microalloy elements in sample B is high, this result was not far from our expectations. The reason is that these elements significantly helped increase the amount of BH by forming Nb and V carbides, dissolving the carbides, and raising the dissolved carbon at 800 °C. The engineering stress–strain diagrams for sample C at annealing temperatures of 750 and 800 °C with a pre-strain of 8% are shown in Figure 9c,d. As seen in the figure, with the increase in the annealing temperature due to the increase in dissolved carbon in the system, the tensile strength and yield stress increased. In sample C, with the increase in the annealing temperature at the 2% pre-strain, there was no significant change in the BH value. This value was about 32 Mpa at both temperatures. The tensile strength increased at 800 °C. The Lüders strain value is high in both graphs and this is due to the large drop in yield that occurred. However, the amount of the Lüders strain at 800 °C (12%) was less than 750 °C (14.5%). 

**Figure 11:**
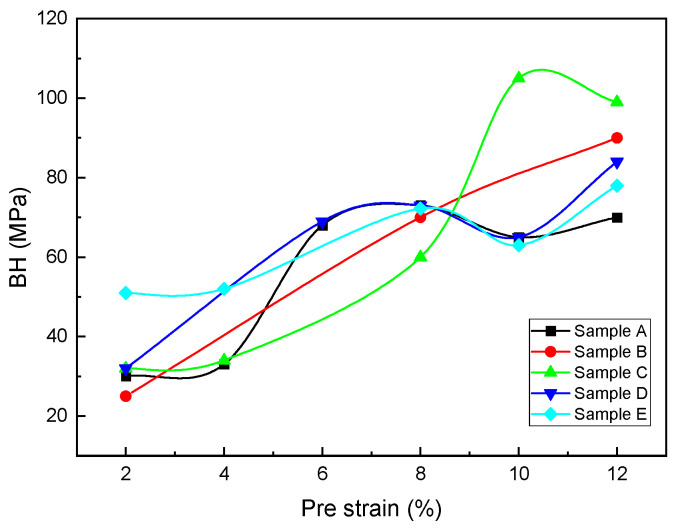
BH variations vs. pre-strains for different samples.

The BH changes in terms of the pre-strains at an annealing temperature of 750 °C are shown in Figure 11. As shown in the figure, until the pre-strain of 8%, the value of BH in sample C is the lowest, followed by the strain of 8%, and the value of BH in sample C is the highest. The highest value of BH in the pre-strain is 10% (105 Mpa). The amount of BH obtained at the pre-strain of 2% in samples A, C, and D is approximately 30 Mpa. At the 8% strain, BH values in samples D and E are almost identical.

**Figure 12:**
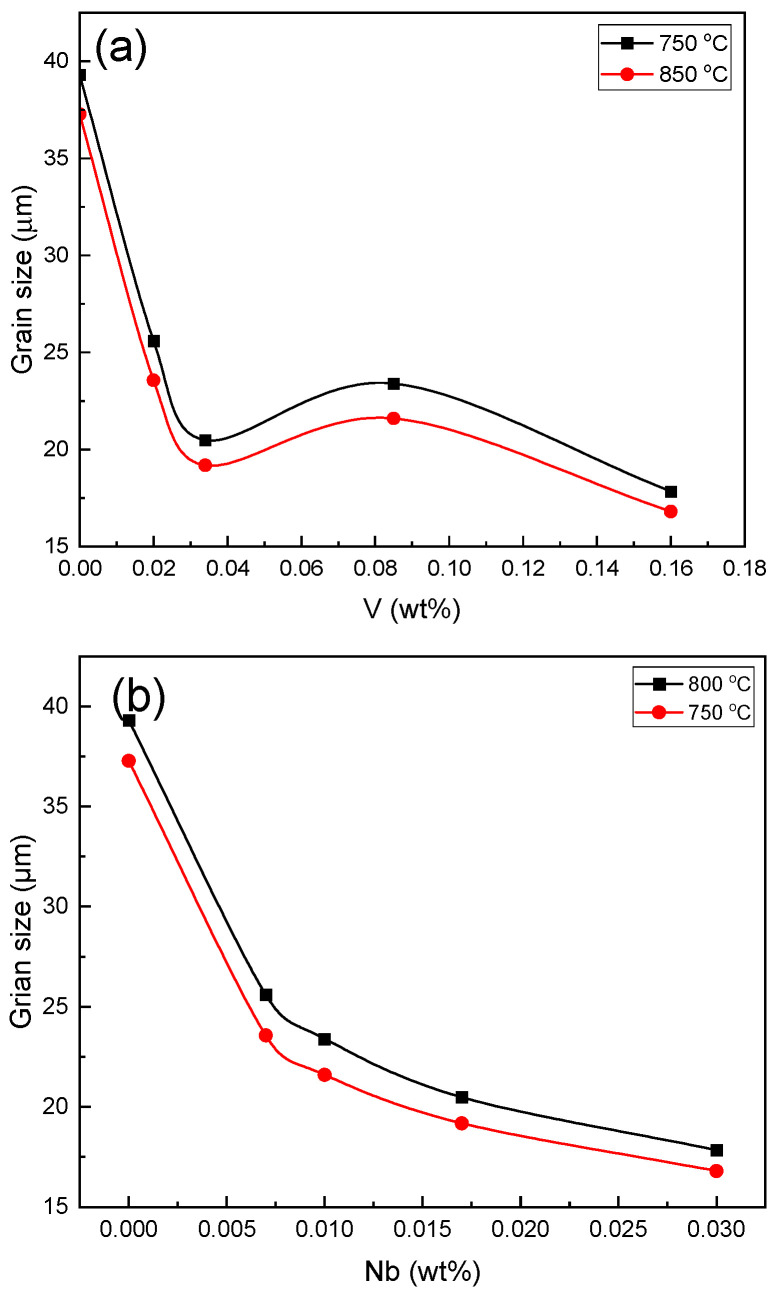
Grain size variations of the different samples: (**a**) samples containing V; (**b**) samples containing Nb.

The grain size changes in the different amounts of Nb content are plotted in Figure 12. As shown in the figure, the grain size decreased with the increasing Nb. This trend can be seen at both the annealing temperatures of 750 °C and 800 °C. However, the changes in grain size with the increase in the V content are smaller, and the grain size decreased except for that of sample C. Therefore, according to the previous research [40], it is concluded that the amount of V did not significantly affect the refinement, and it mostly played a role in reducing the recrystallization annealing temperature and increasing the dissolved carbon of the steel.

**Figure 13:**
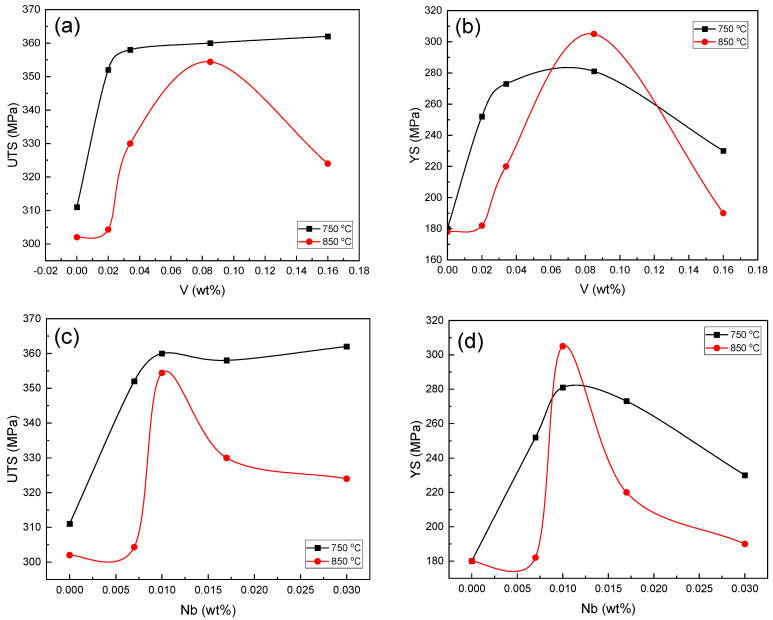
(**a**) UTS, (**b**) YS variations in the different samples containing V; and (**c**) UTS, (**d**) YS variations in the different samples containing Nb.

Figure 13 shows the changes in UTS and yield stress according to the amount of Nb and V contents. According to the figure, sample B with 0.031% Nb and 0.16% V had the highest tensile strength. On the contrary, in samples A and E, in which the Nb percentage was lower, the tensile strength value was lower than that of the rest of the samples. Therefore, it can be justified that the tensile strength increased by increasing the percentages of the Nb and V contents despite the very low carbon. Figure 13b,d show the yield stress changes according to the amount of Nb present in the samples. Sample C with 305 and 281 MPa at two annealing temperatures of 750 and 800 °C, respectively, had the highest yield stress. Moreover, sample C had the maximum yield stress in the samples containing V. The lowest value of the yield stress was related to sample A, which did not contain the Nb and V microalloy elements.

**Figure 14:**
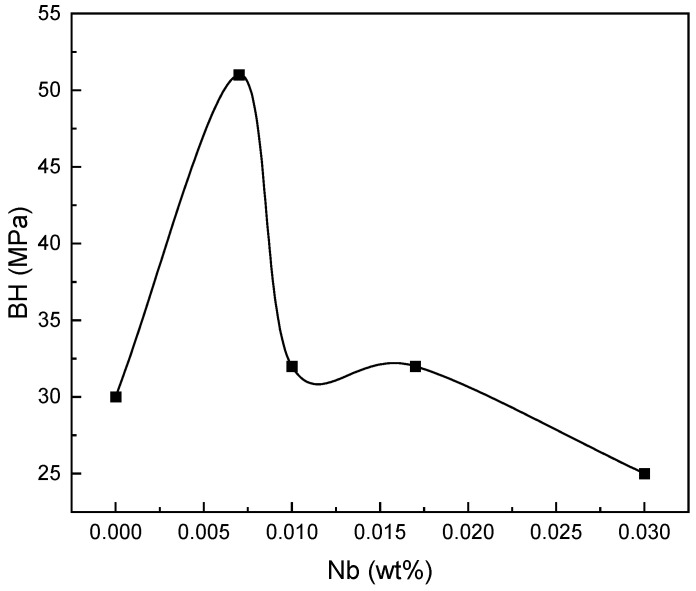
BH variations vs. Nb content.

According to Figure 14, sample E had the highest BH value at the 2% pre-strain. Samples C and D had the same BH values, and samples B and A had the lowest BH values. The reason for the higher BH in sample E is related to having the lowest amount for the Nb/C ratio. The closer the Nb/C ratio is to one, the more dissolved the carbon is and the more significant is its effect on the amount of BH [31,42]. Precipitates act as preferred sites for recrystallization. In steels that are coiled after hot rolling at low temperatures, the beginning and end of recrystallization is performed at high temperatures. Furthermore, the duration of the recrystallization is longer than that of coiling at high temperatures. In steels whose Nb/C ratio is ~0.8, recrystallization is performed faster. Moreover, after continuous annealing, the size of the ferrite grains is larger in steel whose Nb/C ratio is 0.8 [31,42,43]. The lower the Nb/C ratio, the higher the amount of BH due to the increase in the amount of dissolved carbon in the steel.

## 4. Conclusions

Bake hardening is an essential part of special steel production. Studies in this field have focused on steels under homogeneous yielding, but until now, no studies have been conducted for the phenomena that occur for steels under heterogeneous yielding. For this purpose, the effect of adding Nb and V alloying elements on the strength of ultra-low carbon (ULC) steel after the bake hardening was investigated.

1. The steel produced with 0.004% carbon and various compounds of niobium and vanadium increased the yield stress due to the BH process.
2. By examining the microstructure of the samples using SEM, coarse carbides have been observed in the microstructure. By studying and examining these deposits, it was concluded that they are primary carbides that are not completely dissolved in the steel during the homogenization stage. Considering that the homogenization temperature is almost the same as the dissolution temperature of Nb carbides, the duration of the process should be considered longer to complete the homogenization operation.
3. Using the vanadium microalloying element instead of the titanium microalloying element is valuable because the precipitates created at a lower temperature are dissolved, and the amount of dissolved carbon in the system is increased. As a result, the annealing process is performed at a lower temperature.
4. The ratio of Nb/C is important for the hardening properties, and the lower this ratio is, the more dissolved the carbon in the system is, and the hardening properties are increased.
5. Increasing the recrystallization annealing temperature from 750 to 800 °C, in addition to increasing the grain size, increased the amount of dissolved carbon atoms, and as a result, the amount of BH increased.
6. According to the study of the microstructure and grain size of sample B, it is observed that sample B has the smallest grain size. Considering that sample B has the highest amount of Nb, this proves the effect of Nb on grain size.
7. The general conclusion is that the amount of BH obtained by the steel samples investigated in this research that contained Nb and V microalloy elements was higher than that of the titanium element. In addition, it should be kept in mind that the carbon content of the steels investigated in this research was about 0.004% ultra-low carbon steels, while the steels investigated by most researchers are low-carbon steels.

## Figures and Tables

**Table 1:** Chemical composition of the samples with different V and Nb contents.

Sample	Chemical Composition (wt%)										
	C	Si	Mn	P	S	Mo	Cu	V	Nb	Cr	Fe
A	0.004	0.02	0.14	0.011	0.017	0.01	0.01	-	-	0.001	Bal.
B	0.004	0.02	0.14	0.011	0.016	0.01	0.01	0.16	0.031	0.003	Bal.
C	0.004	0.018	0.14	0.011	0.015	0.01	0.01	0.085	0.01	0.003	Bal.
D	0.004	0.01	0.1	0.011	0.015	0.01	0.01	0.034	0.017	0.003	Bal.
E	0.004	0.01	0.1	0.011	0.015	0.01	0.01	0.02	0.007	0.003	Bal.

**Table 2:** Hardness and strength of the sample after hot/cold rolling.

Sample		Hardness (HV30)	Yield Stress (MPa)	UTS (MPa)	Elongation (%)
Hot-rolled	B	155	240	370	47.6
	E	122	220	234	67.6
Cold-rolled	A	242	190	818	2.24
	B	298	205	944	3.28
	C	277	202	852	3.12
	D	257	200	846	3
	E	277	203	855	2.8

**Table 3:** The results of tensile tests after annealing.

Sample	Annealing Temperature (°C)	YS (Mpa)	UTS (Mpa)	Elongation (%)	Grain Size (µm)
A	650	230	333	43.68	23.9
	750	180	311	45.44	37.28
	800	178	302	47	39.3
B	650	415	543	18.04	16.38
	750	230	362	41.36	16.88
	800	178	302	44.8	17.84
C	650	315	395	33.6	18.8
	750	281	360	36	21.6
	800	305	354.4	36	23.4
D	650	295	378	35.48	17.58
	750	273	358	38.12	19.18
	800	220	330	41.6	20.48
E	650	352	428	28.12	16.93
	750	252	352	36.88	23.57
	800	182	304.3	49.72	25.6

**Table 4:** The results of tensile test after BH process and annealing at 800 °C.

Sample	Pre-Strain	Flow Stress (Mpa)	YS (Mpa)	UTS (Mpa)	Elongation (%)	Uniform Strain (%)	Lüders Strain (%)	BH (Mpa)
A	4	278	315	347.7	33.32	17.24	2.5	37
	10	312	368	369.9	14.36	4.17	1.74	56
	12	272	358	396	26.6	17.8	2.8	86
B	2	200	251	332.2	34.8	27.5	2.6	51
	8	281	338	361.7	17.84	12	2.4	57
C	2	245	276	334.3	30.68	24.5	12	31
	8	299	374	408.7	16.72	13	7	75
D	8	281	365	399.8	30.16	22.4	5.6	84
	12	296	400	434.2	25	19.5	5.4	104
E	8	251	301	340.9	24	16.5	2.8	50
	12	309	390	413.1	23.28	14.4	3.3	81

## Data Availability

Not applicable.
